# Breastfeeding Practices During COVID-19: A Narrative Article

**DOI:** 10.7759/cureus.30588

**Published:** 2022-10-22

**Authors:** Mohit M Raghuwanshi, Lokesh M Vaishnav, Swarupa Chakole

**Affiliations:** 1 Community Medicine, Jawaharlal Nehru Medical College, Datta Meghe Institute of Medical Sciences, Wardha, IND; 2 Public Health Sciences, Jawaharlal Nehru Medical College, Datta Meghe Institute of Medical Sciences, Wardha, IND; 3 Community Medicine, Jawaharlal Nehru Medical College, Datta Meghe Institute Of Medical Sciences, Wardha, IND

**Keywords:** septic, artificial feeds, pandemic, postnatal care, covid-19, breastfeeding

## Abstract

COVID-19 was declared a pandemic because of the rapid rise in cases worldwide. Since then, it has altered the ordinary lives of people around the globe. The surge in the pandemic also questioned breastfeeding practices. As breastfeeding is one of the most critical steps toward the wellness of the newborn and maternal health, whether to follow this practice with a child born COVID-19 positive or in the case of suspected infection in the mother was also questioned. There was little information and awareness on the influence of COVID-19 on breastfeeding and postnatal care of newborns; as a result, this situation created havoc and confusion about which processes were to be carried out and how. Thus, this article examines the supporting data and correct procedures to carry out while breastfeeding newborns born during the pandemic.

For the collection of evidence, searches were conducted using PubMed and Web of Science along with multiple data published on the websites of the World Health Organization (WHO) and Ministry of Health and Family Welfare, Government of India (MoHFW) between the period March 2020 to March 2022. Articles suggested significant changes in the hospital policies, such as disallowing visits to the mother or baby and changes in the mentality of mothers where a few mothers breastfed their newborn with all the septic care, like masks and frequent handwashing practices and others discontinued breastfeeding and used artificial feeds for the newborn. Even the WHO guidelines state that the mother should breastfeed the infant with good septic care. However, due to the havoc of the pandemic and miscommunication of the various policies, there was a gap in implementing the correct measures. This article provides insight into the breastfeeding scenario in COVID-19-positive or suspected mothers with COVID-19.

## Introduction and background

The COVID-19 pandemic has led to havoc and extensive loss of human lives and resources. It has raised various questions about the present scenario of the system and management of infectious diseases. The pandemic has caused confirmed cases of about 557,917,904 (till July 15, 2022) and confirmed deaths of approximately 6,358,899 (until July 15, 2022) worldwide [1]. During the pandemic, though there were a lot of data and guidelines for the adult population, essential parts of the population comprising pregnant people and their newborn children were underprivileged, as the data and policies at the time lacked critical data that would have helped to carry out routine procedures. Also, data on the vertical transmission of infection from mother to infant was also lacking [2-6]. There was also concern about the negative impacts of COVID-19 exposure on neonates [7]. With the view of neonates getting infected with COVID-19 from their mothers after birth either through vertical transmission or through breastfeeding, there had been fewer articles or confirmed cases. Still, the chances of transmission have been postulated. Until September 18, 2020, about 16 studies have been published, and nearly half of them possess a positive test for SARS-CoV-2 ribonucleic acid in breast milk [4,8-13].

The pandemic has changed and challenged every aspect of day-to-day life; one of the most influential groups affected significantly was new parents who were affected by the rapidly evolving guidelines, shortage of data, fear of infection, and application of social distancing; significant challenges with which they required mental and physical help [14]. In the United Kingdom, various organizations stressed the security and significance of continuing breastfeeding [15]. The technique of newborn feeding that is most frequently advised worldwide is breastfeeding. However, there are cultural differences in breastfeeding patterns such as higher percentages of exclusive and sustained breastfeeding in low- and middle-income countries, and the use of formula feeding more common in Western Europe, Australia, and North America [16].

In addition to all these issues, lactation and nursing make this an arduous process [17]. However, having confidence in oneself and trusting in one's ability to make decisions is commonly suspended during major infectious disease epidemics like the COVID-19 pandemic. For instance, mothers with COVID-19 may experience clinical barriers to breastfeeding; there are few professional assistance options available to parents in the early postpartum weeks to help them cope with difficult situations, such as when the journey of the young parents is complicated by social isolation practices that impair the social or familial environment [18]. Contrarily, a mother who had not planned to breastfeed her newborn due to her job or any other related issues may do now as a result of the extension of epidemic, which increased the number of mothers at home, and there may be a rise in breastfeeding patterns accordingly [19].

The World Health Organization (WHO) initially published clinical treatment guidelines on COVID-19 on March 13, 2020, which included advice on postpartum care and breastfeeding for mothers and newborns during the hospital stay. Direct nursing, skin-to-skin contact, and living together were all strictly advised by the WHO to limit the risk of COVID-19 transmission, along with other suggested hygienic practices as well as proper nourishment of the newborn [20]. The following article focuses on gathering all the facts from various articles around the globe to showcase the study on the information gap and the role of miscommunicated guidelines in creating havoc; this study also focuses on how one could prevent these mistakes in the future and carry out smooth administration and management of disease on a worldwide level right from the lowest level of clinical care operators.

## Review

Pregnancy during the COVID-19 pandemic is more critical than ever to protect oneself and the baby. There is always an increased risk of severe flu during pregnancy, for which influenza vaccination is given to protect the baby and mother for other vaccinations including COVID-19, one should ask their healthcare providers. Various symptoms experienced, such as fever, cough, pain in the chest, dizziness, severe muscle pain, seizures, difficulty breathing or shortness of breath, and reduced urine output, should seek immediate medical help. Precautions like cleaning hands frequently, keeping at least a 1 m distance from others, wearing a mask, coughing, or sneezing into the bend of elbow or tissue, avoiding touching eyes, nose and mouth frequently, and avoiding public gatherings. Childbirth during COVID-19 was also a big challenge; according to World Health Organization, all women have the right to a safe and positive childbirth experience, whether they have confirmed COVID-19 infection or not. Guidelines also include that a child born COVID-19 positive should be supported by breastfeeding safely and skin-to-skin and rooming practices. COVID-19-positive mothers should follow the following practices during breastfeeding: (1) Practice respiratory hygiene and wear a mask; (2) wash hands before and after touching the baby; (3) routinely clean and disinfect surfaces [20].

Thus World Health Organization respected every pregnant woman and the newborn child as well as their rights to a positive childbirth experience and safe breastfeeding while forming the guidelines. Keeping all the policies in mind there were various difficulties in their implementation as there was a lack of awareness. Also, different studies were carried out throughout the world to increase the knowledge on how and by what mechanism the virus can be transmitted from the mother to newborn and how can one prevent it. Various difficulties in applying the standard guideline and the study of different modes of transmission are addressed in this article.

Difficulties in setting up a unique and intense care unit for pregnant COVID-19 patients

Breastfeeding during COVID-19 brought a stigma along with the risk of transmission of the infection from a positive or suspected mother to the newborn. In the beginning, there had not been sufficient data for the conclusion or evidence that there occurred transmission of COVID-19 infection through breast milk.

In a study in India in April 2020, there was difficulty in developing various facilities for suspected pregnant women with COVID-19. As mentioned in the survey of the management of the first patient with confirmed COVID-19 in pregnancy in India: from guidelines to frontlines, the challenges faced can be divided into three that are (1) local standard operating procedures to be adopted, (2) setting up a triage area, and setting up the site for the patient with suspected COVID-19 infection, and (3) managing patients with confirmed COVID-19. The solution to the various challenges mentioned above is given in the table below in Tables 1-3 [21].

**Table 1:** Setting up a Triage Area A list of the numerous difficulties encountered in establishing an obstetric hospital for COVID-19 patients. From Sharma et al. [21]

Area: close to or beyond the current labor room	Because a maximum of patients might visit there.
A special group	There should be a separate and special team other than the on-call team for the triage area
PPE	Arrangement of proper PPE to be made available as per the standards for the screening area.

**Table 2:** A Special Area to be Set up for Suspected Patients A list of the numerous difficulties encountered in establishing an obstetric hospital for COVID-19 patients. From Sharma et al. [21]

Determine a suitable site with separate entry and exits and air conditioning, and isolation	Like the outpatient department, which is non-functional at movement
Separate manpower for the area that includes nursing staff and resident team	A separate team is to be formed on a shift basis for the special area
Setting up operation theatre's location, anesthesia, oxygen supply	Location: minor operation rooms in the outpatient departments; anaesthesia: to be moved from another department; oxygen supply: through cylinders

**Table 3:** During the Procedure (A List of the Numerous Difficulties Encountered in Establishing an Obstetric Hospital For COVID-19 Patients) From Sharma et al. [21]

Given that everyone on the crew was wearing PPE, It was challenging to communicate with the outside team from the surgical team.	There needs to be a special team to manage the communication between the team.
Formats for consent are not standardized	Consent request formats are not standardized.
There are several difficulties with moving patients into and out of delivery suites. After moving a patient, the route should be meticulously cleaned.	Solutions may come from research into economical transportation options.

Risk of infection passing from pregnant COVID-19-positive patients to their babies during nursing

Breast milk is an essential and valuable part of the life of an infant or a newborn as it is rich in various antimicrobial factors along with antibodies like IgA. Also, it includes multiple viruses and bacterial neutralizing factors [22,23]. For many infections like human immunodeficiency virus (HIV), breastfeeding is a mode of transmission where breastfeeding is contraindicated, contrary to diseases like hepatitis B and hepatitis C. There were a few articles that described the presence of ribonucleic acid (RNA) of the virus in the breastmilk, and a few also suggested the mode of vertical transmission; however, if the presence of RNA were examined using the reverse transcription-polymerase chain reaction (RT-PCR) method, there would have been the possibility of contamination of the sample during collection [24-26]. SARS-CoV-2 RNA is rarely discovered in breast milk samples from women diagnosed with SARS-CoV-2 infection. No signs of culture were present of SARS-CoV-2 from any sample, and the longitudinal follow-up showed that even when it is found, it is not possible to be the cause of infection for the breastfed baby as viral RNA was very briefly present [27].

Nutritional factors in breastmilk have an antiviral effect on SARS-CoV-2

**Figure 1:**
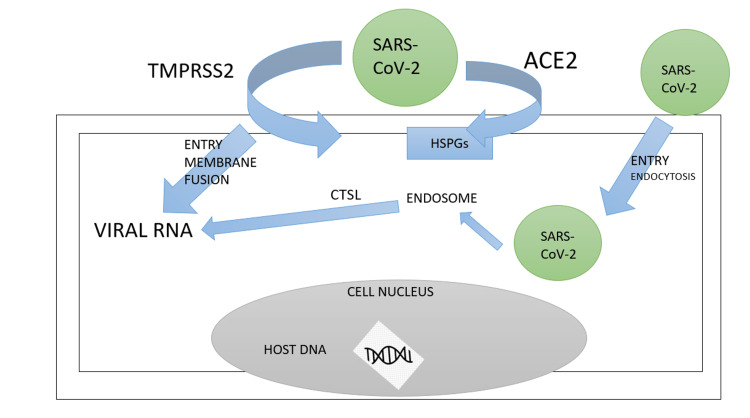
The process through which SARS-CoV-2 enters the host cell From Pang et al. [33] The above figure is created by the authors. ACE2: angiotensin-converting enzyme 2; TMPRSS2: transmembrane protease serines 2; HSPG: heparan sulfate proteoglycans

SARS-CoV-2 binds to heparan sulfate proteoglycans (HSPG) and entries through either of the two pathways, either by high-affinity receptor angiotensin-converting enzyme 2 (ACE2) or in cells with high expression of transmembrane protease serines 2 (TMPRSS2). SARS-CoV-2 enters the cell through membrane fusion-mediated infection after TMPRSS2 cleaves S protein into S1 and S2 subunits further binding with S2 [28-31]. There is a deficient expression of receptor angiotensin-converting enzyme 2 (ACE2) in human females, their reproductive organs, and mammary glands, so there are very few chances of SARS-CoV-2 infection there [32]. Breastmilk contains a variety of substances that can block the activity of SARS-CoV-2, making it impossible to separate the pathogenic SARS-CoV-2 from breastmilk where protein, lactoferrin, mucins, and secretory immunoglobulin A are considered for the various protective mechanism of breast milk from the virus (by inhibiting replication, inhibiting viral particle replication) (Figure 1) [33].

Low risk of vertical transmission

Vertical transmission refers to the infection from mother to child during the antepartum or intrapartum periods through the placenta, or by the contact of the body fluids during birth or breastfeeding during post-pregnancy care [34]. A receptor for entry of SARS-CoV-2, angiotensin-converting enzyme 2 (ACE2), is present at the maternal-fetal interface. Thus the infection can reach the placenta with the virus theoretically. But various practical studies suggest the placental transmission of the infection is usually less than 5% [35]. The survey of Jafari et al. [36] stated that about 12% of the placental specimen, 5.6% of amniotic fluid, and 6% of umbilical cord swabs were detected for the RNA of SARS-CoV-2, indicating a deficient transmission of infection through these routes. Precautions to be taken while breastfeeding during COVID-19 by the World Health Organisation and Ministry of Health and Family Welfare, Government Of India are as follows:

The mother should be counseled about the benefits of initiation and continuation of breastfeeding for her and the baby, even if she is suspected or positive for COVID-19 [20]. Also, hospital staff should consider the practice of rooming-in and skin-to-skin contact, including kangaroo mother care, immediately after birth. If the mother has COVID-19 on suspicion or after testing positive, take additional measures. To stop the infection from circulating, she should also follow the practices like wearing a mask, regular and frequent washing of hands, using a tissue to sneeze, and then disposing of it [37].

Discussion

Exclusive breastfeeding has many useful lifelong effects on the newborn. It helps in various aspects of life, such as reducing mortality, providing protection from different infectious diseases, and developing the immunity of the neonate. Along with all these biological factors, it helps build social factors such as bonding between mother and neonate and attachment [38]. Recognizing breastfeeding as the most important event in a neonate's life, our article focuses on the effect of the pandemic of COVID-19 on breastfeeding. This study found that there occurred delay in the initiation of breastfeeding or the expressed breastfeeding in the mothers who were positive for the infection rather than the one who was normal. This late initiation of the process can be attributed to the strict isolation and quarantine measures; this was also seen due to the recommendation of the Chinese national guidelines that breastfeeding should be suspended for women found to be COVID-19 positive and to have given birth [39]. Different studies showed positive as well as negative impacts on breastfeeding during COVID-19. Positive impacts were the extension of maternal leaves, which lengthened the time mothers had with their babies. Negative impacts were high in number, like the restriction of social and professional support, especially in the case of new or young parents, which also had psychological impacts on them, and delay in the initiation of breastfeeding [40]. The question that arises is how we make difference moving forward. It is unlikely in such situations of a pandemic where strict isolation is followed to give face-to-face interventions, rather, proper formation of the guidelines with deep study of the disease, with knowledge of all its pathophysiology and pathogenesis should be followed and equal importance should be given in making facilities available to every patient at all levels of health care facilities. Information in today’s world can spread and be made available worldwide using online or telephone support systems.

This research does have limitations. Online research collection data is used in collaboration with the above-mentioned information. However, there may have been the exclusion of the data from the group who could not access the internet. This article does not address different complications of breastfeeding such as sore nipples, reduced breastmilk production, or any complications of the baby latching onto the breast. This review contains information in English only, which is why it does not contain the whole spectrum of information.

## Conclusions

There was great uncertainty about how to proceed with breastfeeding throughout the COVID-19 outbreak, from the medical practitioner to the specific population, especially the new parents. As mother and child health care is one of the most vital parts of global development, this part was left like a question before us all during the rapid spread of infection. In the beginning, there were significantly less data available and very little research on this topic, so there was no conclusion and a difference in the practices followed globally. On the one hand, there was fear of the spread of infection to the newborn, and on the other hand, there was the loss of an essential part of that infant's life. There were guidelines and roles to play in how to breastfeed a baby.

Further research found that there are relatively few chances of spreading the virus through vertical transmission or through breast milk to the newborn because of the molecular structure of the receptor and the presence of various chemicals and proteins which prevent the spread of infection from the mother to the fetus. Breastfeeding has been shown to boost baby survival rates and lower the risk of newborn intestinal and respiratory illnesses. SARS-CoV-2 can only very rarely be transmitted vertically and through breastfeeding. The International Children's Emergency Fund, the World Health Organization, the United Nations, and most countries allow mothers with COVID-19 to interact with and nurse their children while adhering to the appropriate sanitary rules, such as wearing face masks, using alcohol to clean surfaces, and gaining written informed consent from parents. However, with each pandemic wave, guidelines for COVID-19 patients who are pregnant have constantly been changing.
